# Mobile Robot Path Planning Based on Time Taboo Ant Colony Optimization in Dynamic Environment

**DOI:** 10.3389/fnbot.2021.642733

**Published:** 2021-03-01

**Authors:** Ni Xiong, Xinzhi Zhou, Xiuqing Yang, Yong Xiang, Junyong Ma

**Affiliations:** ^1^College of Electronics and Information Engineering, Sichuan University, Chengdu, China; ^2^The Second Research Institute of Civil Aviation Administration of China, Chengdu, China; ^3^Civil Aviation Logistics Technology Company Limited, Chengdu, China

**Keywords:** path planning, mobile robot, ant colony algorithm, dynamic environment, time taboo strategy

## Abstract

This article aims to improve the problem of slow convergence speed, poor global search ability, and unknown time-varying dynamic obstacles in the path planning of ant colony optimization in dynamic environment. An improved ant colony optimization algorithm using time taboo strategy is proposed, namely, time taboo ant colony optimization (TTACO), which uses adaptive initial pheromone distribution, rollback strategy, and pheromone preferential limited update to improve the algorithm's convergence speed and global search ability. For the poor global search ability of the algorithm and the unknown time-varying problem of dynamic obstacles in a dynamic environment, a time taboo strategy is first proposed, based on which a three-step arbitration method is put forward to improve its weakness in global search. For the unknown time-varying dynamic obstacles, an occupancy grid prediction model is proposed based on the time taboo strategy to solve the problem of dynamic obstacle avoidance. In order to improve the algorithm's calculation speed when avoiding obstacles, an ant colony information inheritance mechanism is established. Finally, the algorithm is used to conduct dynamic simulation experiments in a simulated factory environment and is compared with other similar algorithms. The experimental results show that the TTACO can obtain a better path and accelerate the convergence speed of the algorithm in a static environment and can successfully avoid dynamic obstacles in a dynamic environment.

## Introduction

In mobile robot navigation, global path planning has always been one of the research hotspots. At present, the research on path planning of mobile robot in static environment has been relatively mature, bringing many excellent kinds of algorithms. There are some traditional algorithms, such as A* algorithm, artificial potential field method (Rimon and Koditschek, 1992), and Dijkstra. Besides, heuristic optimization algorithms are also among the lists, including genetic algorithm, neural network algorithm (Khan et al., 2020c), particle swarm optimization algorithm, ant colony algorithm (Fan et al., 2003), cuckoo algorithm (Mohanty and Parhi, 2016), bug algorithm (Khan et al., 2020a), and so on. However, all kinds of algorithms are more or less limited to algorithm deficiencies. Compared with the traditional gradient descent algorithm, the metaheuristic algorithm performs better in convergence speed, and global optimization ability and hence is widely used in trajectory planning (Khan et al., 2020b), prediction, resource scheduling, and other fields. Ant colony algorithm with its good robustness, positive feedback, and parallel computing ability has been widely used in robot path planning and achieved good results.

In Zhou et al. (2013), an improved ant colony optimization (ACO) algorithm is proposed. By modifying the initial environment pheromone and state transition probability, the search deadlock can be eliminated. The combination of deterministic search and random search can reduce redundant paths. Jiang et al. (2019) adopt a method to make the initial pheromone uneven, which can reduce the blind search path of ants, cut down the running time of the algorithm, and improve the convergence rate of the algorithm. In Luo et al. (2019), the improved pheromone updating strategy was used to update the excellent path and punish the poor path; meanwhile, the upper and lower limits of pheromone were set. In Liu and Zhang (2016), the simulated annealing algorithm is added to the process of simulated annealing ant colony algorithm formed by ant colony algorithm, and it is applied to path planning to solve the local optimal problem caused by premature ant colony algorithm. However, the working environment of mobile robot is always dynamic, such as intelligent factory, hospital, supermarket, and so on. Therefore, it is a difficult problem to avoid obstacles successfully and make quadratic optimal path planning after encountering dynamic obstacles. In Li et al. (2019), an inertial positioning strategy is proposed to enable the robot to predict the position of the target in advance. From the predicted position, the robot path is generated by cubic spline interpolation, and then the improved particle swarm optimization algorithm with random positive feedback factor in speed update is used to optimize the path. As it is very important to track and predict dynamic obstacles in dynamic environment, Ferguson et al. (2008) proposed that if a dynamic obstacle runs on a straight road, it is likely that it will continue to travel along the same straight line in the future. Li et al. (2017) constructed a prediction model to avoid obstacles. In Kim et al. (2017), a trajectory prediction method based on occupancy grid and neural network is proposed. In Qu and Huang (2015), different obstacle avoidance strategies were set up to avoid dynamic obstacles when the mobile robot met with dynamic obstacles. The size of dynamic obstacles is considered in Xu et al. (2019) on the basis of Qu and Huang (2015). The above literatures predict that the dynamic obstacles are moving on a straight line without considering the change of the speed of the dynamic obstacles. There are unknown dynamic obstacles in the dynamic environment. Ant colony algorithm also has the problems of slow convergence speed and poor global search ability.

This article proposes a novel, improved ACO algorithm using time taboo strategy, namely, time taboo ant colony algorithm, which aims to solve the path planning problem of mobile robot in dynamic environment. It utilizes the adaptive initial pheromone uneven distribution to reduce the blindness of ants in early path finding. And, the problem of deadlock is solved by the rollback strategy; pheromone preferential limited update is adopted to reduce pheromone redundancy. The above improvements are designed to improve the convergence speed and global search ability of the algorithm. The corresponding strategies, mechanisms, and prediction models are proposed to avoid dynamic obstacles. Based on the improvement, strategy, and prediction model mentioned above, the dynamic obstacle avoidance of mobile robot is effectively realized and verified by simulation.

## Ant Colony Optimization

ACO is a heuristic global optimization algorithm in evolutionary algorithm. In the process of searching the path, ants release pheromones on the path through which they have passed. The shorter the path is, the higher the concentration of pheromone released will be. Therefore, the following ants will favorably choose the path with highest concentration of pheromone, as a result of which an optimal path can be obtained.

In order to reduce the detour of ants in the process of searching, the algorithm introduces the concept of taboo table. In the process of ant searching, the taboo table is added to the path nodes that have been explored, and the next time ants select the path nodes, optional nodes will exclude those in the taboo table. At the same time, the heuristic function η is introduced, and the following transfer function is used to improve the search efficiency of ant colony. The transfer formula (1) and the heuristic function (2) are as follows:

(1) $$P=\{\begin{array}{ll} \frac{{\left[\tau_{ij}\left(t\right)\right]}^{\alpha }{\left[\eta_{ij}\left(t\right)\right]}^{\beta }}{\sum_{s\in A}{\left[\tau_{ij}\left(t\right)\right]}^{\alpha }{\left[\eta_{ij}\left(t\right)\right]}^{\beta }} & if\;j\in A\\ \;0 & else \end{array}$$

(2) $$\begin{array}{rcl} \eta_{ij}\left(t\right) & = & \frac{1}{d_{j}} \end{array}$$

In the formula,τ_*ij*_ represents the pheromone concentration from the *i*th node to the *j*th node on the feasible path node. η_*ij*_ represents the heuristic value from the *i*th path node to the *j*th path on the feasible path, and its value is the reciprocal of the distance from the *j*th node to the end point, as shown in formula (2), *d*_*j*_ represents the distance from the *j*th node to the end point. *S* represents the current path node. *A* represents the nodes that ants can still choose after removing obstacles and path nodes in the taboo table. α and β represent the importance of pheromone and heuristic pheromone, respectively. According to this formula, we can know that the higher the pheromone concentration is, the higher the heuristic pheromone will be, and the greater the probability of ants selecting the node will be.

At the same time, the pheromone concentration on the map will gradually evaporate with the running time. The updating of pheromone is shown in the following formulas (3) and (4).

(3) $$\begin{array}{r} \tau_{ij}\left(t+\Delta t\right)=\left(1-\rho \right)\tau_{ij}\left(t\right)+\Delta \tau_{ij}\left(t\right) \end{array}$$

(4) $$\begin{array}{r} \Delta \tau_{ij}\left(t\right)=\sum_{k\;=\;1}^{m}\Delta \tau_{ij}^{k}\left(t\right) \end{array}$$

ρ stands for the evaporation coefficient of pheromone. The larger the value is, the more the pheromone evaporates, with its value between (0 and 1). Pheromone evaporation can avoid excessive accumulation of pheromone. Δτ_*ij*_(*t*) represents the pheromone increment from the *i*th node to the *j*th node at time *t*. Its definition is shown by formula (5).

(5) $$\Delta \tau_{ij}^{k}\left(t\right)=\{\begin{array}{c} \frac{Q}{L_{k}}\;P\left(i,j\right)\in P\\ 0\;P\left(i,j\right)\notin P \end{array}$$

In the formula, *K* represents the *k*th ant, *L*_*k*_ represents the total length of the *k*th ant's path, *P* represents the nodes in the path, *P*(*i, j*) represents the path nodes from *i* to *j*, and *Q* represents the pheromone strength. The formula shows that only the ants that have reached to the destination can update pheromones, and the pheromone concentration is inversely proportional to the length of the path. Although the ants in the ant colony do not communicate directly, they communicate through pheromone, an indirect medium, to achieve the goal of optimal path planning.

## Ant Colony Optimization Improvement

Some factors will directly affect the performance of ant colony algorithm, such as pheromone, pheromone updating rule, heuristic pheromone, state transition rule, and deadlock and self-locking of ant colony algorithm. However, the improvement of the initial pheromone is still rigid, which is not conducive to the global search of the algorithm, and the processing of pheromone change caused by the improved deadlock problem is not perfect yet. In this article, pheromone, deadlock and self-locking, and pheromone update mechanism are improved. Based on these improvements, the time taboo ACO (TTACO) is further proposed.

### Adaptive Initial Pheromone Distribution

In the basic ant colony algorithm, the pheromone distribution is quite even; even pheromone distribution may cause ants wander around and backtrack, rendering too-long search time of ants in the early stage of the algorithm, and the length of the search path is increased. In this article, the pheromone near the connection line is added according to the distance from the starting point to the end point, and the pheromone of the node closer to the connection line is higher in concentration. At the same time, in order to prevent the algorithm from falling into local optimum due to the initial pheromone, the algorithm will determine the number of pheromones added according to the proportion of obstacles on and near the starting and ending lines. The formula is as follows:

(6) $$\{\begin{array}{c} \tau =\tau_{0}+\vartheta C\\ \vartheta =\mu \varepsilon \end{array}$$

In the formula, τ is the pheromone of ant colony algorithm, τ_0_ is the basic pheromone, ϑ is the adaptive parameter, and μ is the distance between the node and the line; the closer the distance is, the greater the value will be, with the range of the value being [0, 1]. When the distance between the obstacle and the connection exceeds a set value, the value of *u* is taken as zero. The closer the grid is to the line, the higher the pheromone concentration will be. Because of the existence of obstacles, ε will decrease, resulting in the decrease of the overall pheromone. According to the connection between the starting point and the end point, it is highly probable that the optimal path is the path near the line, and the condition of the obstacle near the line will hinder the degree of the optimal path near the line. Therefore, the uneven distribution of the initial pheromone is conducive to the ants' searching for the best path in the early stage.

### Solving Deadlock and Self-Locking Problems

When the ants explore the path in the early stage, because of the restriction of the map environment and the taboo table, the individual ant may fall into the dilemma of finding nowhere to go before reaching the end point. It can be divided into deadlock and self-locking. Inspired by Dai et al. (2019), this article uses a rollback strategy to solve the deadlock and self-locking problems and divides the taboo table into a global taboo table and a local taboo table. The global taboo table records information of deadlock location, and the local taboo table, inherited from the global taboo table, records the information of the ant's walking path node and the self-locking position node. The ant chooses the path according to the local taboo table. When the ant has a deadlock problem caused by the environment, the ant is made to retreat two steps, and the place where the ant falls into the deadlock will be added to the global taboo table to reduce the possibility that the ant falls into the same deadlock again. When the ant is self-locked because of its walking path, the ant is made to go back two steps. Different from the deadlock, the position where the ant self-locking occurs will be added to the local taboo table to prevent the ant from repeatedly walking into the same self-locking node.

### Pheromone Update Rules

Because of the introduction of the rollback strategy, all ants can successfully find the end point. Although the search ability of the algorithm is improved, the increase in the number of ants that successfully reach the end point leads to pheromone redundancy. So pheromones of ants will be updated only with the shortest path and shorter path to reduce the redundancy of pheromones on the map. The number of ants updated with the pheromone is determined by the scale of the map. The larger the map is, the more ants can get pheromone updates.

### TTACO

The basic ant colony algorithm introduces a taboo table, but its use of the taboo table is limited to preventing ants from walking nodes that have been already walked. In order to further improve the algorithm's global search capability, algorithm convergence speed, and the ability to avoid dynamic obstacles, this article proposes a time taboo strategy: In the case of path redundancy or detection of dynamic obstacles, ants will be prohibited from accessing certain path nodes for a certain period of time until path redundancy is avoided, or dynamic obstacles are successfully avoided, and then the prohibition is canceled in a specific way. And based on this strategy, a three-step arbitration method and an occupancy grid prediction model are proposed to improve the algorithm's global search ability and the ability to avoid dynamic obstacles.

#### Three-Step Arbitration Method

**Figure 1:**
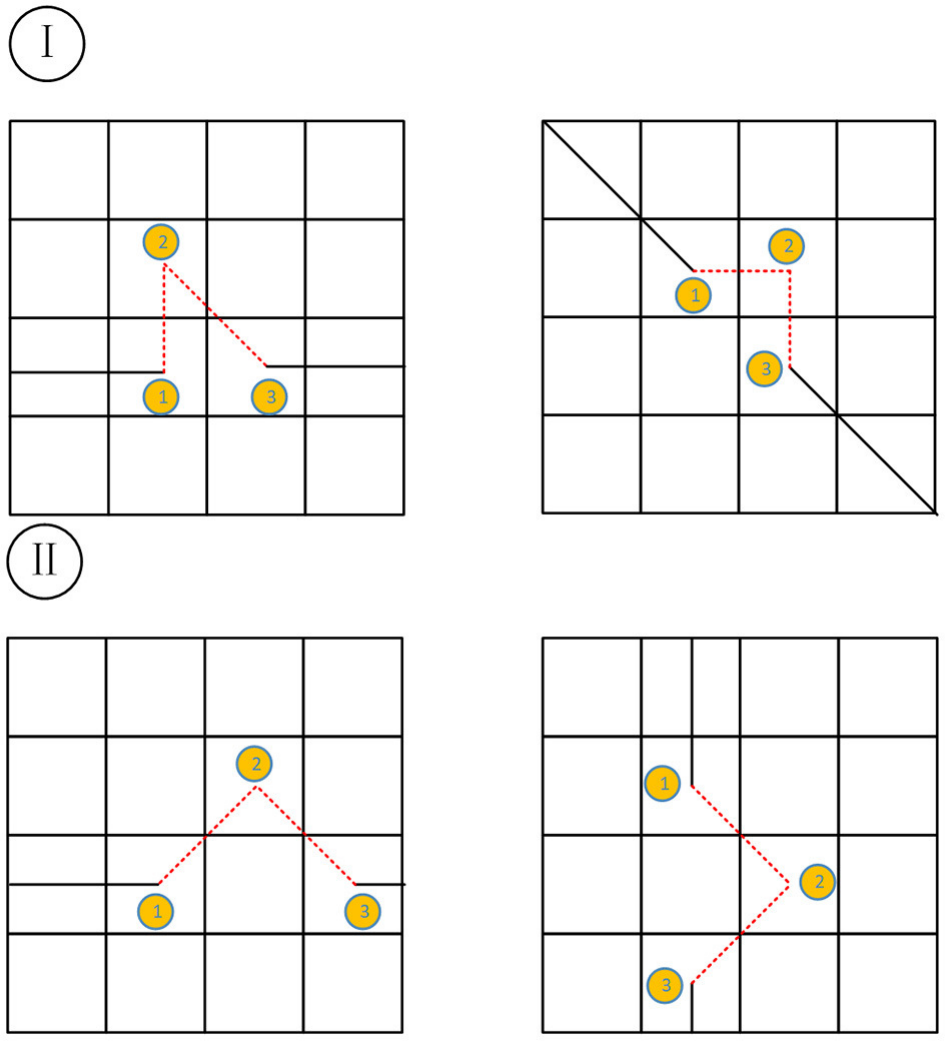
Schematic diagram of path redundancy.

In the crawling process of ants, there will inevitably be path redundancy, as for which this article divides it into two situations: one is redundancy situation I, and the other is redundancy situation II. The two cases are shown in Figure 1, where the top two grids show situation I, and the bottom two are situation II.

When the redundancy situation occurs, the path is divided into three steps. The first step is before the situation occurs, the second step is when the situation occurs, and the third step is after the situation occurs. The specific judgment method is as follows. *S*_12_ and *S*_13_ are used to represent the distance between the first step and the second or third step, respectively. The specific determination rules are shown in Table 1 below.

**Table 1:** Determination rules.

**Path condition**	**Determination formula**
Situation I	*S*_13_ <2
Situation II	*S*_13_ = =2&&*S*_12_!=1

When it is determined that the second step is redundant, the algorithm will record the node where the problem occurs. When the ant crawls to the first step, the algorithm will temporarily add the path node of the second step to the taboo table, so that the ant cannot choose the second step. In this way, the algorithm will accelerate the convergence speed and improve global search capability. The crawling paths of ants in the early stage are more chaotic, and more redundant ones will also occur, so the method is disabled in this period. The algebra that specifically enables the method is shown in formula (7):

(7) $$\begin{array}{r} K_{s}=mod\left(MM,5\right) \end{array}$$

*K*_*s*_ is the algebra of activating the method, and MM is the scale of the map. The larger the scale of the map is, the later the method will be activated. In order to enable a suitable number of ants to invoke the method and avoid multiple redundant path nodes caused by an individual ant to trigger the method at the same time, which will lead to redundant path, and having referred to the cuckoo algorithm (Luan et al., 2015), the following trigger mechanism is proposed.

1. This method is only enabled from the generation of ants crawling a relatively stable path.

2. The use of this method or not will be determined by the probability before each ant searches.

3. When the ant using this method encounters a redundant node, the use of this method or not will be once again determined at the node according to the probability.

#### Occupancy Grid Prediction Model

**Figure 2:**
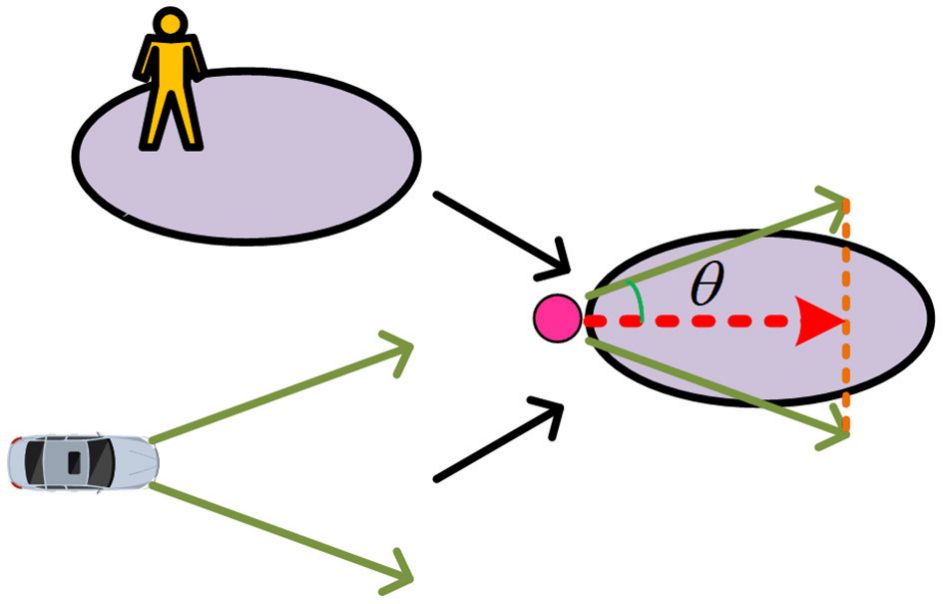
Schematic diagram of dynamic obstacle movement area.

Highly dynamic scenes have brought great challenges to robot path planning. In Zhao et al. (2020), the classical method of detecting and planning at the same time cannot meet the requirement of safely avoiding dynamic obstacles. Kim et al. (2017) use a prediction method based on occupancy grids, where the scene is divided into grids, and the occupancy grids are used to represent the possible locations of dynamic obstacles in the future. Ferguson et al. (2008) propose that if a dynamic obstacle is driving on a straight road, it is very likely that it will continue to drive along the same straight line in the future. Rummelhard et al. (2014) proposes a new grid-based collision risk prediction method. Li et al. (2017) take the mobile robot's kinematics model as the algorithm's trajectory prediction model. Riosmartinez et al. (2013) propose a pedestrian area based on Gaussian distribution. Two individual areas of different sizes are constructed with pedestrians as the center, with the area in front of the pedestrian being lager, assuming that our dynamic obstacles are most likely to be moving mechanical vehicles and pedestrians, as shown in Figure 2.

The kinematics of mobile robot is described in Boukens et al. (2019) and Keyvan et al. (2020). The motion model of a dynamic obstacle can be expressed by the following differential equation, where (*x, y*) is the position, θ is the angle of the dynamic obstacle velocity, *v* is the velocity, *k* is the curvature, and ω is the angular velocity. It can be seen from the formula that when the velocity is constant, the greater the curvature is, the greater the value of θ will be, and the greater the angular velocity ω of the dynamic obstacle will be.

(8) $$\{\begin{array}{c} \dot{x}=vcos(\theta )\\ \dot{y}=vsin(\theta )\\ \dot{\theta }=kv \end{array}$$

(9) $$\begin{array}{r} \omega =vk \end{array}$$

**Figure 3:**
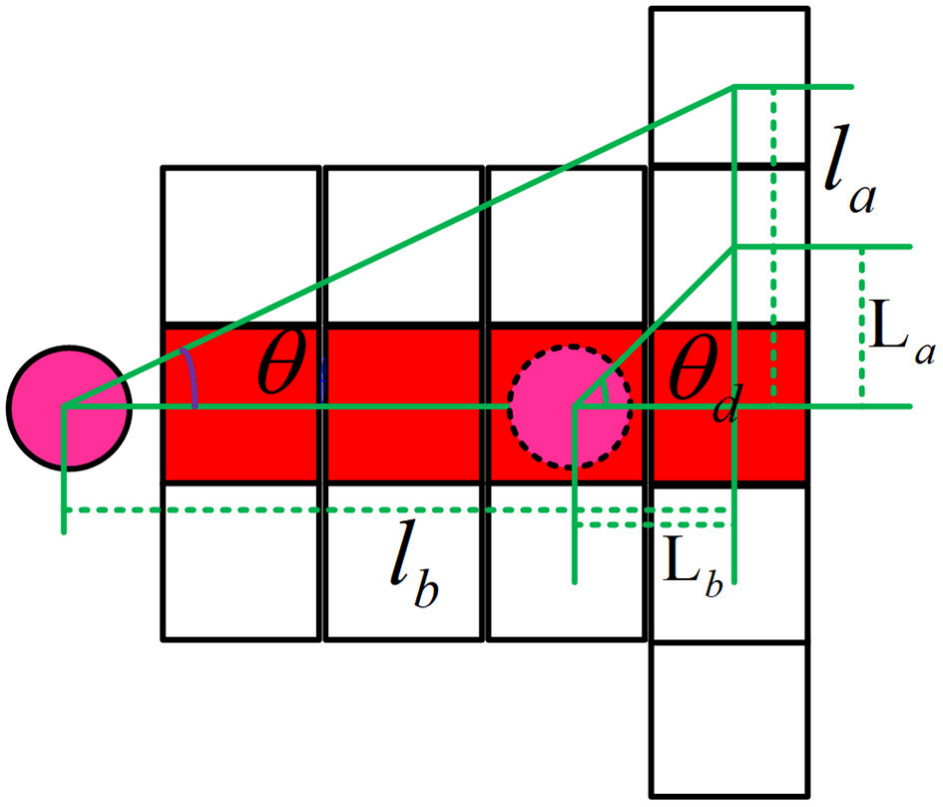
Schematic diagram of dynamic obstacle prediction angle.

It is assumed that the dynamic obstacle has two predicted steering angles, a safe steering predicted angle and an emergency steering predicted angle. Let θ in the above formula be the safe steering prediction angle, that is, the angle at which a slight path deviation of dynamic obstacles may occur is considered, and the safe steering angle is considered only when starting to predict. θ_*d*_ is the emergency steering predicted angle, which represents the angle when the emergency steering of the dynamic obstacle occurs, and each step of the prediction has an emergency steering angle. The schematic diagram is shown in Figure 3.

In the figure, θ is the predicted angle of safe steering, and θ_*d*_ is the predicted angle of emergency steering, and its formula is as follows:

(10) $$\begin{array}{r} \theta =\text{arctan}\left(\frac{l_{a}}{l_{b}}\right) \end{array}$$

(11) $$\begin{array}{r} \theta_{d}=\arctan\left(\frac{L_{a}}{L_{b}}\right) \end{array}$$

In the above formulas, *L*_*a*_ is the offset distance in the vertical direction when the dynamic obstacle makes an emergency turn, and *L*_*b*_ is the distance in the horizontal direction when the dynamic obstacle is making an emergency turn. *l*_*a*_ is the vertical distance when the dynamic obstacle slightly deviates from the path, and *l*_*b*_ is the horizontal distance when the dynamic obstacle slightly deviates from the path. And the angle of θ is set as (0, 26.12°) and the angle of θ_*d*_ (0, 45°).

**Figure 4:**
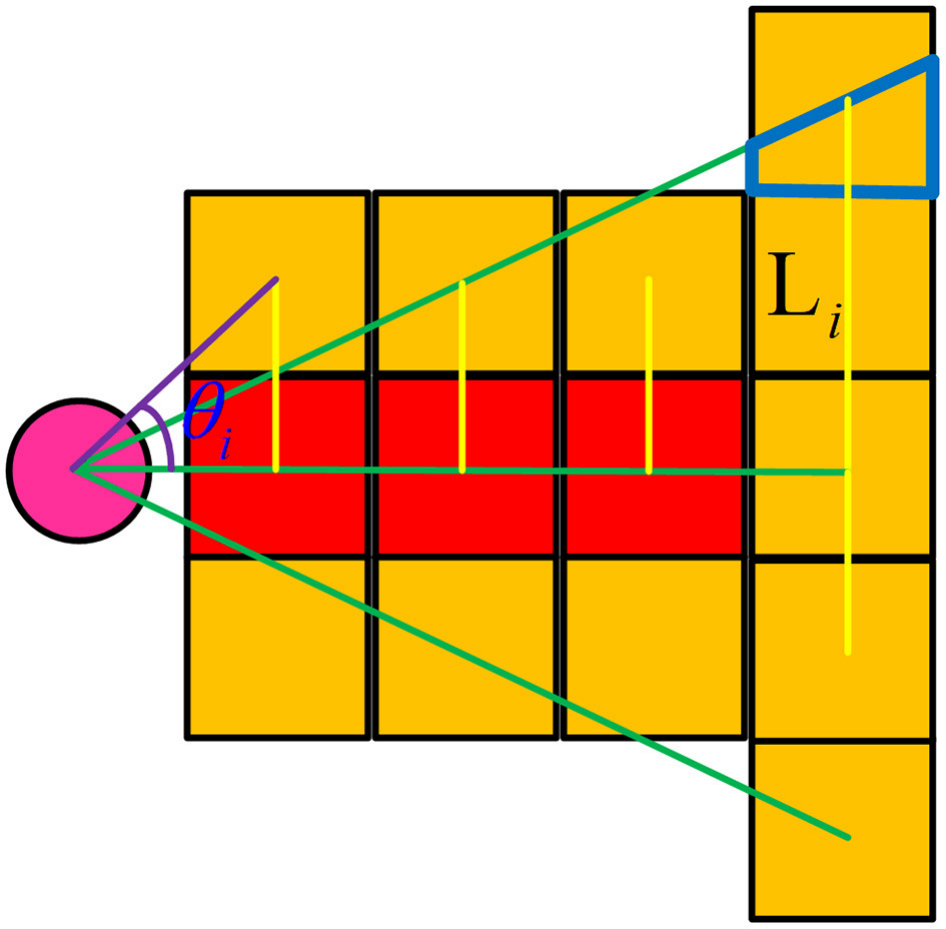
Schematic diagram of prediction of dynamic obstacle occupancy grids.

Figure 4 shows a schematic diagram of path nodes that have been marked as occupied. According to the previously set angle, further specific analysis of whether a path node is affected by dynamic obstacles is determined by the following formula, where *m* is the ratio of the affected area of a grid to the whole grid area. As shown in Figure 4, the ratio of the area of the blue trapezoid to the area of the whole grid and the value is affected by θ. *L*_*i*_ is the vertical distance from the center of the *i*th grid to the velocity direction of the dynamic obstacle, as shown by the golden yellow straight line in Figure 4. γ is an adaptive parameter whose value is shown in formula (13). θ_*i*_ is the angle between the center of the *i*th grid and the speed direction of the dynamic obstacle, which includes θ_*d*_. When the value of *f* is > 0.5, it means that the grid is affected and will be tinted yellow.

(12) $$\begin{array}{r} f=\frac{\gamma }{L_{i}}+m \end{array}$$

(13) $$\begin{array}{r} \gamma =sin\left(\theta_{i}\right) \end{array}$$

**Figure 5:**
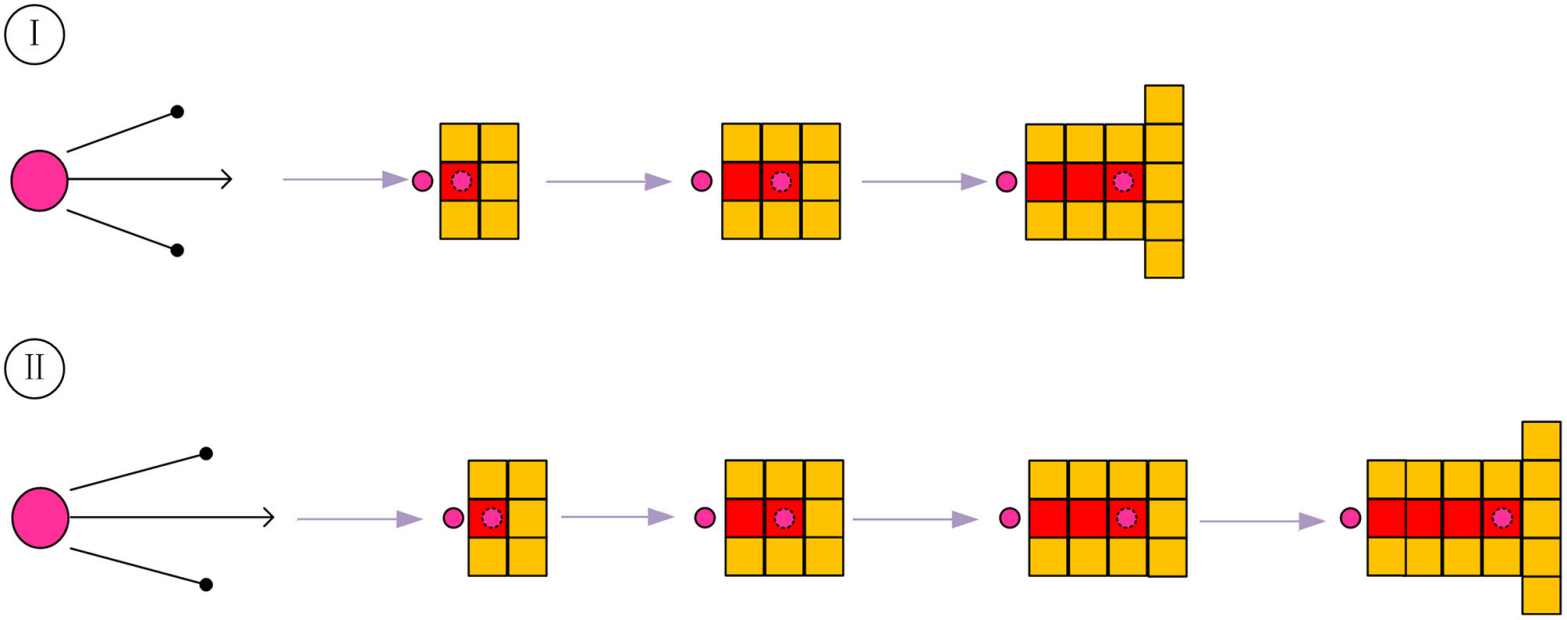
Schematic illustration of step-by-step occupancy grids prediction for dynamic obstacles with different angular velocities.

Combining the possible movement of the dynamic obstacle and the dynamic obstacle movement model, the following occupancy grid prediction model is proposed. When the mobile robot detects a dynamic obstacle, it is predicted that the future path of the dynamic obstacle will continue to follow the current speed direction of the dynamic obstacle, as shown in the red grids in Figure 5 below, and a certain probability of deviation to the original speed direction, as indicated in yellow grids. The longer the path is, the higher the probability of deviating from the red path may be. We set that the predicted footprint of the dynamic obstacle is related to the angular velocity of the movement of the dynamic obstacle. The forecast is shown in Figure 5 below. *v* is the current speed of the dynamic obstacle and remains unchanged. The upper part of Figure 5 shows the step-by-step occupancy grid prediction map when the angular velocity ω of dynamic obstacle movement is large, and the lower part of Figure 5 shows the step-by-step occupancy grid prediction map when the angular velocity ω of dynamic obstacle movement is small. The solid circle in the figure represents the location of the dynamic obstacle at the current time, and the dotted circle represents the location of the dynamic obstacle at the future time.

According to the original path and the predicted path of the dynamic obstacle, it should be judged whether it is necessary to call the model, and the time point to call the occupancy grid prediction model should be calculated. Suppose the mobile robot is walking along the original path, when the distance between the mobile robot and the dynamic obstacle is less than the safe distance, the occupancy grid prediction model is called in the first two-unit time when it is less than the safety distance. After the time and location of the occupancy grid prediction model are obtained, the nodes affected by the occupancy grid prediction model are recorded. The formula for the distance between the mobile robot and the dynamic obstacle is as follows, where (*x*_*a*_, *y*_*a*_) represents the position of the mobile robot, (*x*_*b*_, *y*_*b*_) represents the position of the dynamic obstacle, *S* represents the safety distance, and let *S*_0_ be the judgment limit for safety distance.

(14) $$\begin{array}{r} S=\sqrt{{\left(x_{a}-x_{b}\right)}^{2}+{\left(y_{a}-y_{b}\right)}^{2}} \end{array}$$

Occupancy grid prediction model predicts the distance of dynamic obstacle based on the track length of dynamic obstacle when the distance between mobile robot and dynamic obstacle is less than safety distance. As shown in the following formula (15), *F* (*s*) represents the cumulative length of the dynamic obstacle track length *S*_*b*_ when the distance between the mobile robot and dynamic obstacle is less than the safety distance, and the rounded-up value is the predicted distance of the algorithm for dynamic obstacles.

(15) $$\begin{array}{r} F\left(s\right)=\underset{S<S_{0}}{\sum }S_{b} \end{array}$$

(16) $$\begin{array}{r} S_{T}=F\left(s\right) \end{array}$$

The rules for calling the occupancy grid prediction model are as follows:

1. When the mobile robot detects an unknown dynamic obstacle, it is predicted based on the occupancy grid prediction model to obtain the initial predicted position, time, and occupied grids of the dynamic obstacle.

2. According to the position, time, and occupied grids obtained in the first step, the path nodes that may be affected will be added to the local taboo table. The TTACO will be run, and every time a moment has passed, the path nodes that the dynamic obstacle has passed and are no longer possible to pass are removed from the local taboo table until all the occupancy grids do not exist in the local taboo table.

3. We only consider the possible path nodes during the period of time when the dynamic obstacle has an impact on the mobile robot. Therefore, if the dynamic obstacle deviates from the red predicted line during this process, we repredict and repeat the above processes 1, 2, and 3.

**Figure 6:**
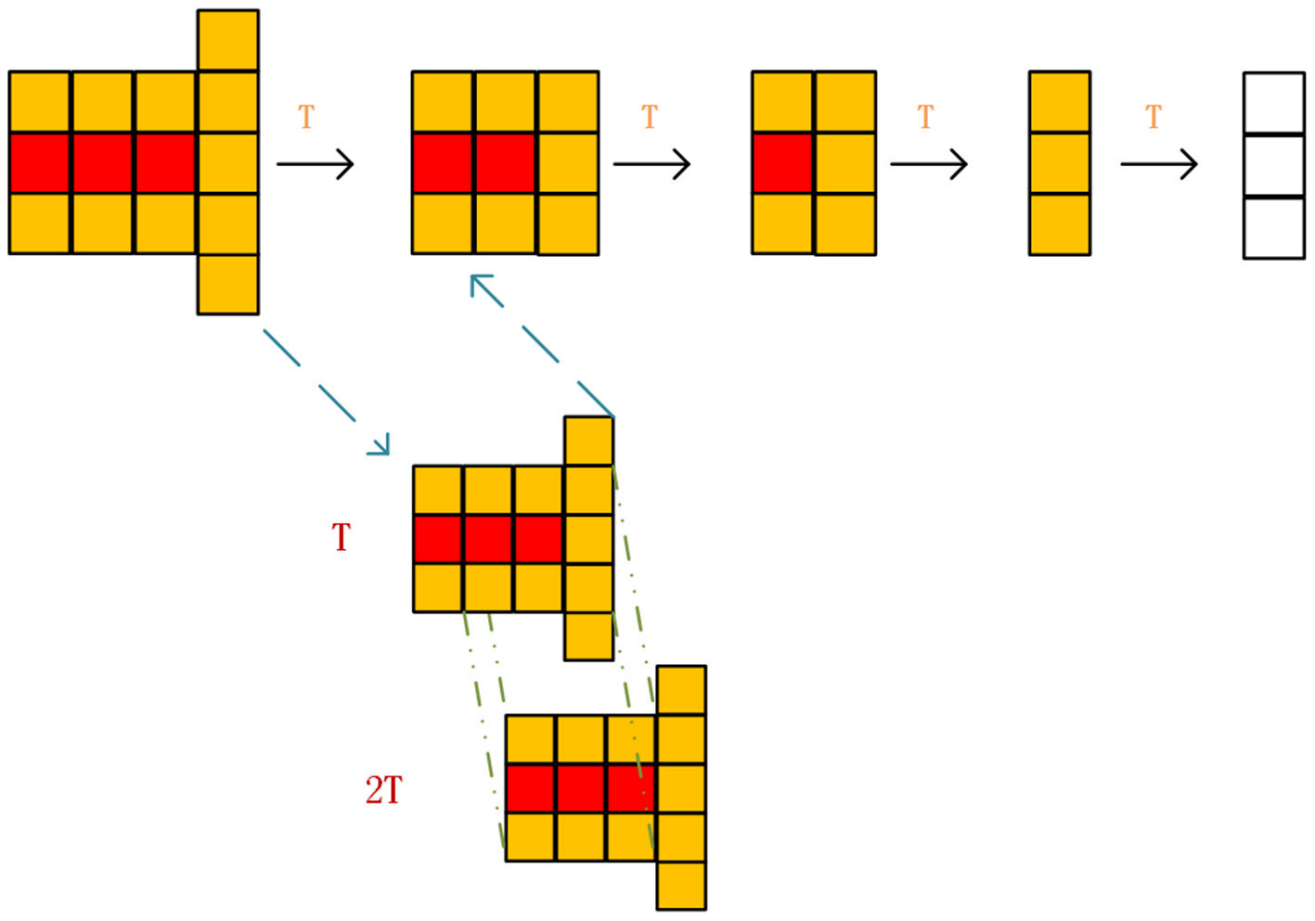
Schematic diagram of misplaced overlap elimination of occupancy grids.

When removing path nodes from the local taboo table, we adopt dislocation overlap elimination. To be specific, we overlap the occupancy grid prediction maps of two moments to obtain the occupancy grid map of the next moment. The schematic diagram is shown in Figure 6 below.

The predictive model is used to predict the possible path nodes of dynamic obstacles in the future moments, study the influence of the possible path nodes of dynamic obstacles on the path of the mobile robot, and give the mobile robot enough time to meet the real-time requirements of the algorithm in the high dynamic environment.

## Dynamic Obstacle Avoidance

### Two Cases of Dynamic Obstacle Avoidance

Dynamic obstacle avoidance is divided into two situations: “encounter” and “chase.” Such division is based on the angle between the speed direction of the mobile robot and the dynamic obstacle speed. When the angle is > 90°, the “encounter” happens, and when it is ≤ 90°, the “chase” occurs. In both cases, the occupancy grid prediction method is used to avoid obstacles. However, when the mobile robot is in a chasing situation with a dynamic obstacle and as the distance is getting closer and closer, it cannot safely pass through. That is, in the TTACO, when the movement of the dynamic obstacle reaches the last predicted ending position, the ant still has not avoided the dynamic obstacle, and the occupancy grid prediction model is called again. If a better path cannot be obtained after calling the model again or several times, the next-generation ants of the TTACO will choose a safe position according to the occupancy grid prediction model and wait for a period of time when avoiding obstacles.

### Ant Colony Information Inheritance Mechanism

When using the occupancy grid prediction model for dynamic obstacle avoidance, in order to improve the calculation speed of the algorithm, we put forward the ant colony information inheritance mechanism. The initial pheromone and taboo table of ACO are determined as follows:

(17) $$\begin{array}{rcl} Tua_{d} & = & Tua+Tua_{s} \end{array}$$

(18) $$\begin{array}{rcl} TABU_{d} & = & TABU_{s} \end{array}$$

Among them, *Tua*_*d*_ is the initial pheromone when the algorithm is called to avoid dynamic obstacles. *Tua* is the original ant colony pheromone matrix, and the values in the matrix are all constant C. *Tua*_*s*_ is the pheromone matrix left by the global ACO algorithm after the upper and lower limits are optimized. *TABU*_*d*_ is the global taboo table that calls the ant colony algorithm when the mobile robot avoids dynamic obstacles, and *TABU*_*s*_ is the global taboo table obtained after the global ACO algorithm runs, and it contains all deadlock position information. Through the inheritance mechanism of the above initial pheromone and the global taboo table, the running speed of the algorithm can be accelerated.

### Algorithm Flow

The pseudocode of this algorithm is shown in Table 2. *TABU*_*s*_ is a local taboo list; *allow*_*k*_ is the list of optional nodes; and *TABU*_*lock*_ is the deadlock taboo list. And the computational complexity of the algorithm is O (n ^∧^ 3). The robot first performs global path planning according to the TTACO and then detects whether there are unknown dynamic obstacles and dynamic obstacle information through its sensors. If there are dynamic obstacles, the occupancy grid prediction model will be used to predict based on the information of the detected dynamic obstacles, and the TTACO is called to replan the path after the ant colony pheromone inheritance mechanism is activated.

**Table 2:** Description of TTACO for solving path planning.

**Algorithm** TTACO **Begin** Create grid environment Adaptive initial pheromone distribution according to formula (6) **Repeat** **for** each ant k **do** Trigger the three-step arbitration taboo method based on probability Add the grids involved in the Occupancy grid prediction model and three-step arbitration taboo method to *TABU*_*s*_ **if** grid i ϵ *allow*_*k*_ **then** **if** grid i ϵ *TABU*_*lock*_ **then** Rollback **end if** According to formula (1) and (5) select next grid j Update taboo **end if** Pheromone preferential limited update for each iteration **Until** Meet the iteration end condition **Return** best grid serial number **END**

## Simulation Experiment

In order to verify TTACO's algorithm convergence speed, global search ability, and capability to avoid dynamic obstacles, a simulation experiment of dynamic obstacle avoidance was carried out in a 30 × 30 simulated factory environment. In order to further verify the performance of the algorithm, it was compared with the algorithms produced by similar articles. The computer performance parameters for the simulation are Intel Core i5-6300HQ processor with a main frequency of 2.30 GHz, a memory size of 8G, the running system of Windows 10, and the simulation software of MATLAB.

### Simulation Experiment in Dynamic Environment

**Figure 7:**
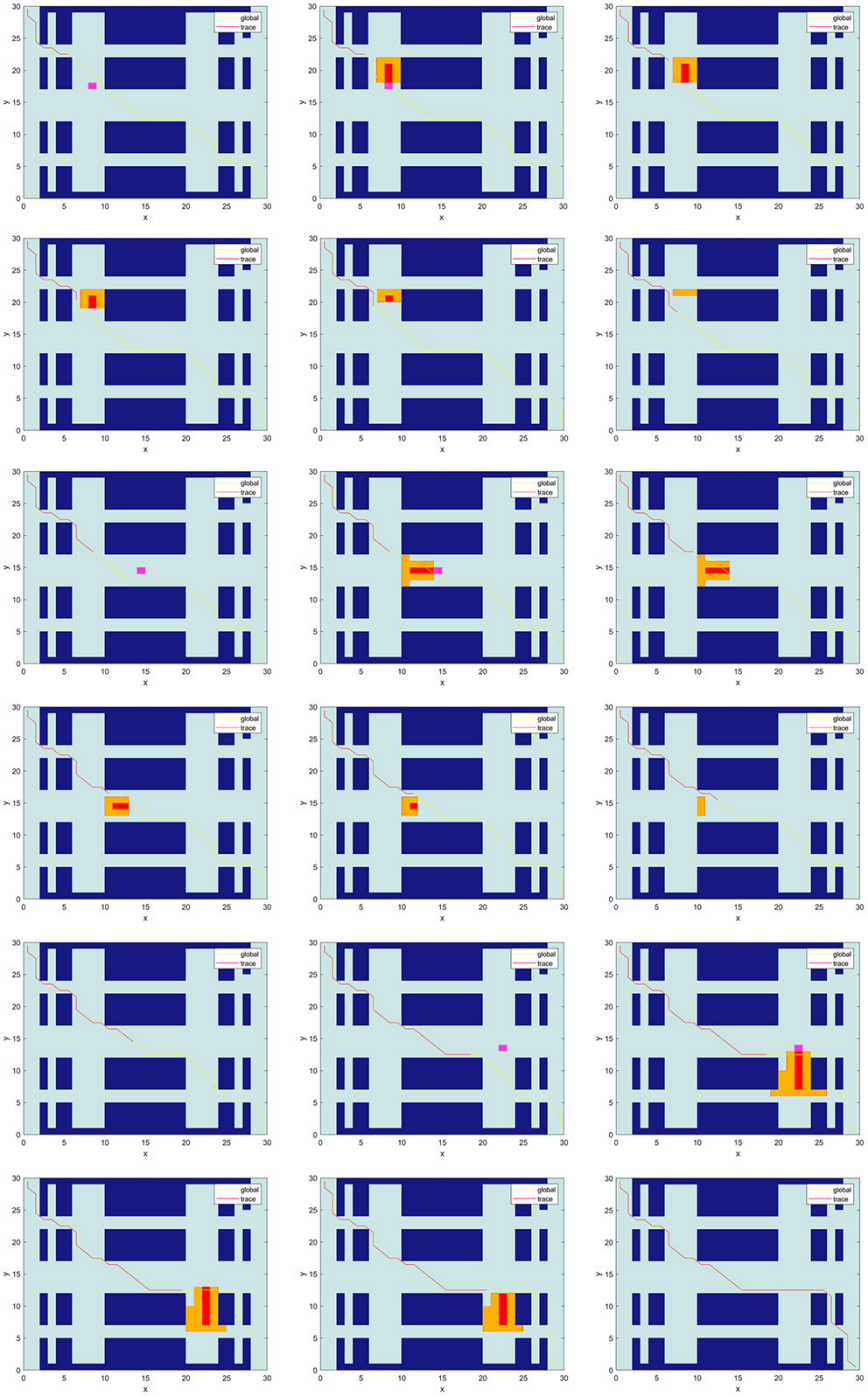
Dynamic obstacle avoidance simulation in Environment 1.

The following simulation is to verify the path planning ability and dynamic obstacle avoidance ability of TTACO in dynamic environment. As shown in Figure 7, Environment 1 is a simulated 30 × 30 factory environment, with three dynamic obstacles set up along the way. Obstacle 1 is a dynamic obstacle moving upward at a speed slightly faster than the mobile robot; Obstacle 2 is a dynamic obstacle moving to the left with a slightly slower speed; and Obstacle 3 is a dynamic obstacle moving downward whose speed is the same as that of the mobile robot. The golden path in the picture is the current planned global path, and the red path is the trajectory of the mobile robot after it has walked along the path. The pink path node is the position of the dynamic obstacle when calling the occupancy grid prediction model. The red path node is the predicted walking path for dynamic obstacles. The yellow path node is the path node where the dynamic obstacle may deviate and make a turn. When the mobile robot encounters a dynamic obstacle, the occupancy grid prediction model is called, and the path is replanned. From the results, we can see that the mobile robot first moves forward along the preset path; then, it calls the obstacle avoidance algorithm and occupation prediction model three times and replans the path three times. The first two calls of occupancy grid prediction model have successfully enabled the robot to avoid the obstacles, and the last call also achieved the purpose of obstacle avoidance through changing the path.

### Comparison of Similar Algorithms

In order to further verify the performance of the TTACO algorithm, the following four sets of comparative experiments are designed to compare the algorithm in this article with the algorithms from other articles.

**Figure 8:**
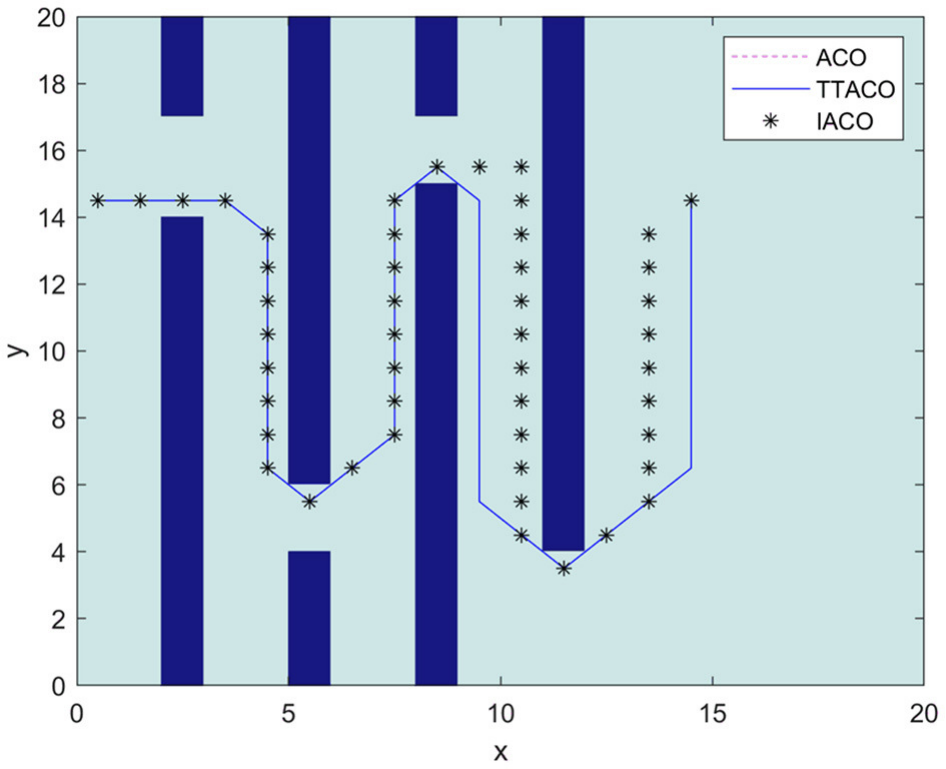
The path planning results of the three algorithms in Environment 2.

Environment 2 is shown in Figure 8 as the environment map in Dai et al. (2019). The improved ant colony algorithm in the literature uses the characteristics of A * algorithm and maximum–minimum ant system and introduces the retraction mechanism to solve the deadlock problem. IACO is used to represent the algorithm in Dai et al. (2019). It can be seen from Figure 8 and Table 3 that both TTACO and IACO successfully find a path; however, the path obtained by TTACO is shorter and converges faster, with the optimal path length being 49.5564 and the average time of iteration 6.7, whereas ACO fails to plan a path.

**Table 3:** Comparison of simulation results in static environment.

**Environment**	**Algorithm**	**Optimal solution of the algorithm**	**Average shortest distances**	**Average iteration times**
E2**^②^**	ACO	–	–	–
	IACO**^①^**	50.7280	51.0605	8.4
	TTACO	49.5564	50.6422	6.7
E3	ACO	31.556	–	27
	IACO1	31.556	–	8
	TTACO	30.97	31.4974	3.5
E4	ACO	47	55.1	80
	IACO2	45.69	–	40
	TTACO	44.12	45.219	9
E5	ACO	47	54.5727	82
	JPACSPF	44.5269	–	9
	TTACO	43.9411	44.234	9

① *: IACO, IACO1, IACO2, JPACSPF represent the algorithms in Xiaoxu et al. (2018), Dai et al. (2019), Zhang et al. (2019), and Ma and Mei (2020)*.

② *: E2, E3, E4, E5 represent the environment maps in Xiaoxu et al. (2018), Dai et al. (2019), Zhang et al. (2019), and Ma and Mei (2020)*.

**Figure 9:**
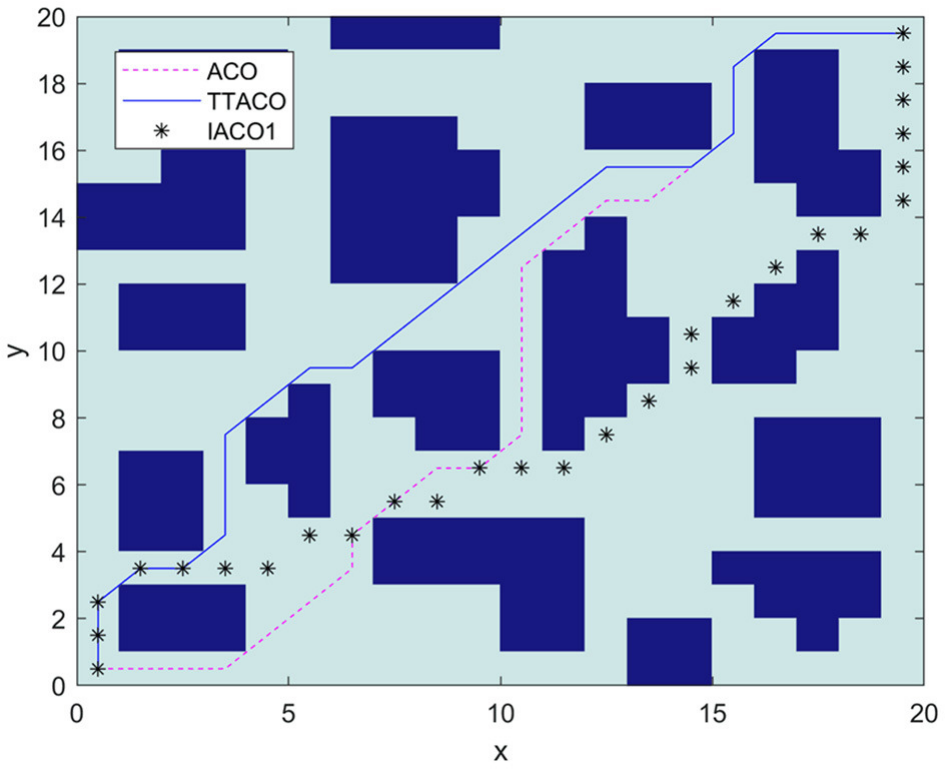
The path planning results of the three algorithms in Environment 3.

Environment 3 is shown in Figure 9 as the environment map in Zhang et al. (2019). In the algorithm, the ant colony is inspired to search the planned path by the improved artificial potential field algorithm. At the same time, the negative feedback channel is constructed by the convergence times, and the size of the environment map is 20 × 20. IACO1 is used to represent the algorithm in Zhang et al. (2019). It can be seen from Figure 9 and Table 3 that the path and times of iteration obtained by the algorithm in this article are better than those given by Zhang et al. (2019) and the ACO algorithm. The optimal path length is 30.97, and the average time of iteration is 3.5.

**Figure 10:**
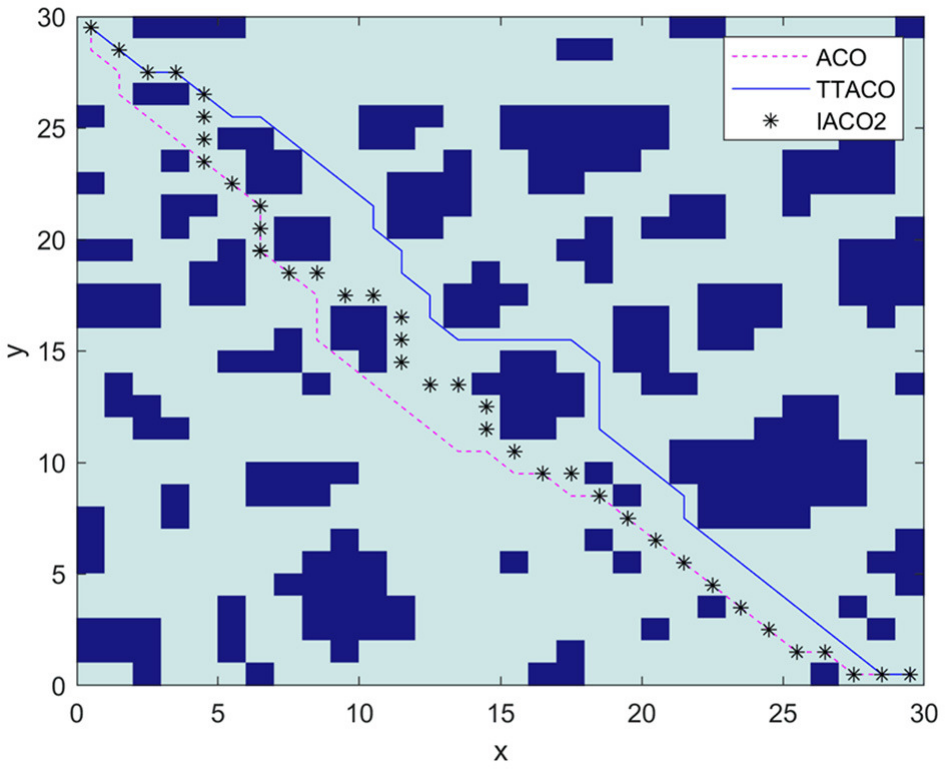
The path planning results of the three algorithms in Environment 4.

Environment 4 is shown in Figure 10 as the environment map in Xiaoxu et al. (2018); this article mainly adopts the strategy of death and rollback for the deadlock problem of ant colony, improves the state transition rules of ant colony algorithm, optimizes the composition structure of pheromone, and replaces the algorithm in the literature with IACO2, with the map size of 30 × 30. From Figure 10 and Table 3, we can see that the path obtained by the algorithm in this article is shorter, which is 44.12, and the average time of iteration is nine times, which is better than the algorithm in Xiaoxu et al. (2018) and ACO.

**Figure 11:**
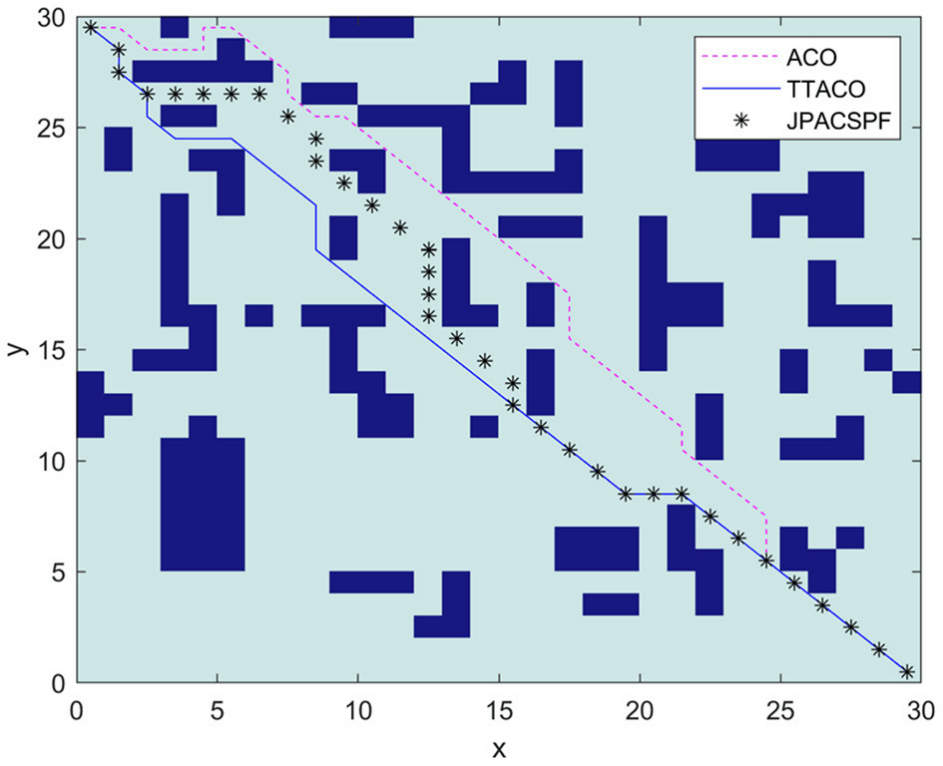
The path planning results of the three algorithms in Environment 5.

Environment 5 is shown in Figure 11 as the environment map in Ma and Mei (2020). The reference algorithm is JPACSPF, which combines the search strategy of ant colony algorithm and jump point search algorithm, and introduces the decreasing coefficient of potential field resultant force. The size of environment map is 30 × 30. Figure 11 shows the paths planned by different algorithms. It can be seen from Figure 11 and Table 3 that the path obtained by the TTACO algorithm is shorter than the path obtained by JPACSPF and ACO, and the average time of iteration of TTACO is better than that of the ACO algorithm. The optimal path length is 43.9411, and the average time of iteration is 9.

By comparing with other algorithms, it can be concluded that both the length of the path and the time of iteration in TTACO are better than those in similar literatures, and the algorithm of this article can get a better path in a shorter time than the ACO.

## Conclusion

This article introduces a novel approach using the TTACO to solve the path planning problem of mobile robots in a dynamic environment. Through the improved adaptive initial pheromone, the death rollback strategy, and the improved pheromone update strategy, the algorithm's convergence speed and global search ability are effectively improved. The three-step arbitration method and occupancy grid prediction model based on the time taboo strategy further improve the algorithm's global search ability and the capability to avoid dynamic obstacles. Through simulation on MATLAB, the experimental results prove that the algorithm can plan a better path in a dynamic environment, so as to realize the navigation of the mobile robot in a dynamic environment. Although the algorithm proposed in this article is novel and has some practical significance, the experimental results need to be further improved, and the details of the article need to be further polished.

## Data Availability Statement

The raw data supporting the conclusions of this article will be made available by the authors, without undue reservation.

## Author Contributions

NX proposed time taboo ant colony optimization (TTACO). XZ provided algorithm improvement guidance and general direction control. YX tested and annotated the algorithm. XY carried out MATLAB simulation in different environments based on the algorithm. JM participated in the preparation of the algorithm and the writing of the paper. All authors contributed to the article and approved the submitted version.

## Conflict of Interest

XY was employed by the company “Civil Aviation Logistics Technology Company Limited.” The remaining authors declare that the research was conducted in the absence of any commercial or financial relationships that could be construed as a potential conflict of interest.
